# Acoustic Change Responses to Amplitude Modulation in Cochlear Implant Users: Relationships to Speech Perception

**DOI:** 10.3389/fnins.2020.00124

**Published:** 2020-02-18

**Authors:** Ji-Hye Han, Andrew Dimitrijevic

**Affiliations:** ^1^Communication Sciences Research Center, Cincinnati Childs Hospital Medical Center, Cincinnati, OH, United States; ^2^Laboratory of Brain & Cognitive Sciences for Convergence Medicine, College of Medicine, Hallym University, Chuncheon, South Korea; ^3^Department Otolaryngology-Head and Neck Surgery, Sunnybrook Health Sciences Centre, Faculty of Medicine, University of Toronto, Toronto, ON, Canada

**Keywords:** acoustic change complex, amplitude modulation, temporal modulation transfer function, cochlear implants, N1

## Abstract

**Objectives:**

The ability to understand speech is highly variable in people with cochlear implants (CIs) and to date, there are no objective measures that identify the root of this discrepancy. However, behavioral measures of temporal processing such as the temporal modulation transfer function (TMTF) has previously found to be related to vowel and consonant identification in CI users. The acoustic change complex (ACC) is a cortical auditory-evoked potential response that can be elicited by a “change” in an ongoing stimulus. In this study, the ACC elicited by amplitude modulation (AM) change was related to measures of speech perception as well as the amplitude detection threshold in CI users.

**Methods:**

Ten CI users (mean age: 50 years old) participated in this study. All subjects participated in behavioral tests that included both speech and amplitude modulation detection to obtain a TMTF. CI users were categorized as “good” (*n* = 6) or “poor” (*n* = 4) based on their speech-in noise score (<50%). 64-channel electroencephalographic recordings were conducted while CI users passively listened to AM change sounds that were presented in a free field setting. The AM change stimulus was white noise with four different AM rates (4, 40, 100, and 300 Hz).

**Results:**

Behavioral results show that AM detection thresholds in CI users were higher compared to the normal-hearing (NH) group for all AM rates. The electrophysiological data suggest that N1 responses were significantly decreased in amplitude and their latencies were increased in CI users compared to NH controls. In addition, the N1 latencies for the poor CI performers were delayed compared to the good CI performers. The N1 latency for 40 Hz AM was correlated with various speech perception measures.

**Conclusion:**

Our data suggest that the ACC to AM change provides an objective index of speech perception abilities that can be used to explain some of the variation in speech perception observed among CI users.

## Introduction

Cochlear implants (CIs) provide electrical stimulation to the auditory nerve that can, in turn, be interpreted by the brain as sound including speech. However, the behavioral benefits gained from CIs vary significantly among recipients; after cochlear implantation, some users achieve highly improved speech perception even in challenging listening situations such as in background noise while others gain very little or no improvement. Nonetheless, the source of the variability in CI performance is still unknown. In general, factors explaining this variation in individual speech perception ability include the bottom-up processing of the auditory periphery to acoustic features (including spectral and temporal information) and top-down cognitive processing at the cortex level (Moberly et al., 2016). However, demographic factors such as age at implantation and duration of deafness merely explain 20% of the variability in CI outcomes (Lazard et al., 2012).

At present, there are no reliable clinically available biomarkers for measuring CI outcomes to help us understand the source of outcome variability. Since the age at implantation can be as low as 1 year of age, developing objective markers is important for assessing pediatric CI users and candidates who have unreliable behavioral responses. Currently used objective measures such as the stapedius reflex, electrically evoked compound action potentials, and electrically evoked auditory brainstem responses have shown poor correlation with speech perception (Abbas and Brown, 1991; Hirschfelder et al., 2012; Lundin et al., 2015). Unlike these peripheral measures, cortical activity measured at the sensory and source levels has nevertheless shown some reliable relationships with behavioral performance in adult CI users in research settings (Han et al., 2016; Gransier et al., 2019).

Psychoacoustic studies have shown that speech perception through a CI relies predominantly on temporal cues because spectral information cannot be effectively delivered due to a limited number of spectral channels and channel interactions (Shannon et al., 1995; Nie et al., 2006). A CI processes the incoming sound, including speech, by applying a series of filter banks to extract the temporal envelope. This envelope then modulates the amplitude of a pulse train that stimulates the auditory nerve. Speech inherently has amplitude modulation (AM) at multiple rates with syllables in the 1–4 Hz range, phonemic information in the 15–50 Hz range and fine structure at higher rates (Rosen, 1992). Therefore, encoding AM is an important feature needed for successful speech perception (Fu, 2002; Edwards and Chang, 2013). Temporal processing is assessed behaviorally by estimating the minimum AM depth needed to detect modulation at various AM rates. The resulting behavioral AM threshold as a function of rate is referred to as the temporal modulation transfer function (TMTF). The shape of the TMTF resembles a low-pass filter with a cut-off frequency near 50–100 Hz (Viemeister, 1979). Compared with normal-hearing (NH) individuals, the TMTF of CI users has a higher overall AM threshold that is more pronounced at higher frequencies resulting in a lower frequency TMTF filter cutoff and subsequently this property is associated with reduced speech perception ability (Won et al., 2011). The ability to detect high-frequency AM (50–300 Hz) is correlated to speech perception in CI users including tone (Luo et al., 2008), consonants (Cazals et al., 1994), and word recognition (Won et al., 2011) and phonemes (De Ruiter et al., 2015). Recently, low frequency AM rate discrimination at 4 Hz shortly after CI activation time was shown to be a predictor of speech perception at 6 months post-activation (Erb et al., 2019).

Previously, we showed that in NH listeners, the N1 cortical evoked potential to AM changes resembles a low-pass filter shape, and the “N1 TMTF” is similar in shape to the behavioral TMTF (Han and Dimitrijevic, 2015). In that study, the N1 acoustic change complex (ACC) to AM changes were smaller at high versus low AM rates. In the present study, we wanted to determine if N1 ACC responses to AM could be elicited in CI users. We hypothesized that the N1 ACC to AM would be related to speech perception ability in CI users.

## Materials and Methods

### Subjects

Ten adult CI users (five females, all self-reported right-handed) were recruited through Cincinnati Children’s Hospital Medical Center according to an Institutional Review Board (IRB)-approved protocol. Their ages ranged from 21 to 84 years (mean age: 50 years). All CI subjects were native speakers of American English based on self-report and had been using his/her CI for at least 1 year prior to enrolling in the study. All CI subjects were postlingually deafened and had severe to profound bilateral hearing loss prior to implantation. They were all bilateral CI users. Table 1 shows the demographic information of the CI users. A composite score based on the average percent scores over a number of speech perception tasks in background noise was the basis for classifying “good” and “poor” performers. There were six good performers with composite speech perception scores ≥50% and four poor users with scores <50%. For the control group (data from a previous study, Han and Dimitrijevic, 2015) 10 healthy NH individuals (six females, mean age = 25.5 years) were recruited. All of them were right-handed and had an audiometric hearing threshold of ≤20 dB HL (hearing level) at octave test frequencies from 250 to 8000 Hz. Participants were compensated for their participation, and informed consent was obtained from all of them prior to participation in the study.

**TABLE 1:** Clinical features of the 10 cochlear implant participants.

CI user	Age (years)	Gender	Stimulated ear	Duration of deafness (year)	CI use (year)	Device/Processor	Processing strategy	Etiology of hearing loss	Composite score	Study group
01	21	M	Left	10	9	Nucleus/CI24RE	ACE	Unknown	78	Good
02	32	F	Left	20	9	Nucleus/CI24RE	ACE	Congenital	26	Poor
03	34	F	Right	33	12	Nucleus/Esprint 22	SPEAK	Hereditary	24	Poor
04	37	F	Right	37	11	Nucleus/CI24RE	ACE	Congenital	16	Poor
05	45	F	Right	37	4	Nucleus/CI512	ACE	Unknown	48	Poor
06	54	M	Left	15	4	Med EI/Opus 2	FSP	Meniere’s Disease	59	Good
07	59	M	Right	11	1	Nucleus/CI24RE	ACE	Noise induced	55	Good
08	63	F	Right	35	3	Nucleus/CI512	ACE	Genetic	55	Good
09	69	M	Left	22	2	Med EI/Opus 2	FSP	Genetic	51	Good
10	84	M	Left	12	10	Nucleus/CI24RE	ACE	Unknown	56	Good

### Behavioral Testing

The TigerSpeech software (House Ear Institute)^1^ was used for the behavioral testing. Consonant and vowel perceptions were measured using a forced-choice paradigm based on a previous report (Fu, 2002). Each of 16 consonants was presented five times (“a/Consonant/a” format, male voice), giving a total of 80 tokens. Similarly, each of 60 vowels was presented five times (“h/Vowel/d” format, male voice), giving a total of 60 vowels. Participants were instructed to indicate which consonant or vowel was heard by choosing the appropriately labeled button on the computer screen, and the performances on the vowel and consonant perception tasks were quantified as percent correct. Sentence and word perceptions were measured using the SPIN (Speech-in-Noise) test (Kalikow et al., 1977). A total of 50 sentences were presented and participants were instructed to repeat each word in the sentence. The number of keywords (the terminal word in a sentence) correctly identified out of 50 was expressed as a percentage. We chose to proceed with electrophysiological testing on the CI side with the higher speech composite score.

The behavioral threshold for AM detection at 4, 40, 100, and 300 Hz was performed in a separate task using a three-interval forced choice with trial-by-trial feedback (Levitt, 1971). The task consisted of presenting three consecutive noise stimuli (1 s duration) one of which was amplitude modulated. The subjected needed to identify which interval had the AM stimulus. The AM depth was varied adaptively. The AM threshold refers to the minimum depth that the subject could detect the AM stimulus (average of the last nine reversals). The process was repeated for all four modulation rates. The depth of AM was defined as the percent ratio between maximum and minimum amplitudes such that 0% had no modulation, 100% was fully modulated (Picton et al., 2003).

### Stimuli

Stimuli were constructed in Matlab using continuous white noise with occasional changes consisting of AM of 1-s duration occurring every 2.2 s on average (the random inter-stimulus interval varied from 1.8 to 2.6 s) and lasting for 1.0 s. Each stimulus with a change in AM as well as the baseline segment was generated from completely novel randomized noise in Matlab. The AM was changed at rates of 4, 40, 100, and 300 Hz. To avoid differences in the overall level that can occur when AM is introduced, the AM portion was multiplied by a factor that equated the root-mean-square of the preceding 1 s (no modulation).

Stimuli were presented in free field through a single speaker at 0° azimuth 1.5 m away from the subject. All stimuli were presented at the most comfortable level for each subject. To estimate the loudness of the stimuli for CI users, an intensity corresponding to loudness level of “7” on an 11-point scale (a 0 to 10: inaudible to too-loud linear scale) was applied (Hoppe et al., 2001). The stimuli were presented to the NH listeners at 70 dB SPL, while the intensity level was variable (70 to 85 dB SPL) for the CI users. The stimuli were calibrated using a Brüel and Kjaer (Investigator 2260) sound level meter set on both A and slow-time weighting with a half-inch free-field microphone.

### Recordings

The electrophysiological data were collected using a 64-channel actiCHamp Brain Products recording system (Brain Products GmbH, Inc., Munich, Germany). Although our CI users were bilaterally implanted, the electrophysiological testing was carried out using one of the CIs while the other was turned off. The side with the higher speech composite score was used for all testing, yielded a total of five on each side. An electrode cap was placed on the scalp with electrodes placed at equidistant locations, the infracerebral cap covering a larger area than is typical in a 10–20 system (Hine and Debener, 2007; Han and Dimitrijevic, 2015). The reference channel was located at the vertex (Cz) while the ground electrode was located on the midline 50% of the distance to the nasion. Continuous data were digitized at 1000 Hz and stored for offline analysis.

### Data Processing

Electrophysiological data were analyzed using Brain Vision Analyzer ver. 2.0 (Brain Products GmbH, Inc., Munich, Germany). Data were high-pass filtered (0.01 Hz) to remove baseline drift and down-sampled to 512 Hz. Visual inspection of the data included the removal of extreme stereotypical artifacts related to subject movement (exceeding 500 mV). Independent component analysis (Delorme and Makeig, 2004) implemented in Brain Vision Analyzer (with an identical algorithm to EEGLAB; Delorme and Makeig, 2004) was applied to reduce ocular and cardiac as well as CI artifacts. This approach decomposed the electroencephalographic (EEG) signal into maximally temporally independent components (ICs). Afterward, when an IC was deemed to be an artifact, its corresponding IC weight was set to zero, thereby minimizing its contribution to the data. In this study, ICs related to the CI were removed when the IC waveform morphology had an abrupt peak within ∼10 ms of the onset/offset of the sound and resembled the AM envelope. The topography of the ICs showed an activation centroid near the location of the CI. Another indication of CI artifact was component energy at the AM modulation frequency. This was performed by computing the frequency spectrum of the IC. The IC with highest energy at the AM rate was removed. This procedure was helpful for CI artifact identification especially at the higher modulation rates (100 and 300 Hz). On average, five ICs or less were removed per CI subject.

After IC artifact reduction, the channel data for the electrodes near the CI were interpolated, the data referenced to average reference, and segmented into epochs −200 to 1500 ms with the AM change stimulus occurring at 0 ms and averaged. The auditory N1 responses, observed by pooling three electrodes in the frontal-central (FC) regions. Manual peak identification occurred over latencies in the 100–200 ms range. Peaks were verified by examining topography and polarity inversions at the mastoid. If no N1 peak was apparent, then this data was considered missing and was not analyzed further.

### Procedures

During the EEG recording, participants were seated in a sound-attenuated booth, asked to watch a silent, closed-captioned movie of their choice, and instructed to ignore the background sounds. A total of 400 trials for each of the four AM change stimulus frequencies were conducted across eight blocks. The total recording time was approximately 1.5 h, and subjects were encouraged to take breaks between blocks.

### Statistical Analysis

Repeated-measures analysis of variance (ANOVA) was used to assess statistical significance for both the psychoacoustics and EEG recordings. Details of the repeated-measures ANOVA factors are given with the results. The non-parametric Mann-Whitney *U* test was conducted to compare differences between the good and poor CI groups, along with *post hoc* analysis using Tukey’s honest significant difference test. Spearman’s rank-order correlation was computed to examine relationships between the speech test scores and the N1 amplitude/latency measures.

## Results

### Psychoacoustics

**FIGURE 1:**
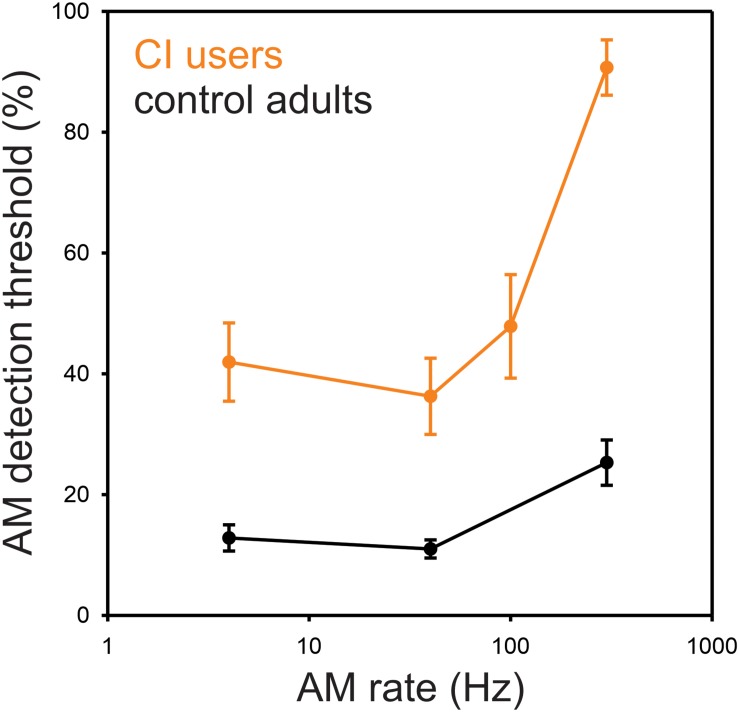
Behavioral AM detection thresholds as a function of AM rate in CI and normal-hearing groups. Shown are the mean detection thresholds across 10 CI and 10 NH participants. Note that the AM detection thresholds were measured at 4, 40, 100, and 300 Hz for CI users, while the thresholds at 100 Hz were not measured for NH participants. The AM detection thresholds in CI users were higher than NH for all AM rates. NH data redrawn from Han and Dimitrijevic (2015).

The minimum AM depth needed for detection of modulation for 4, 40, 100, and 300 Hz was, on average, 44, 37, 49, and 77%, respectively (Figure 1) where greater values indicate poorer performance requiring higher modulation depth for detection. The repeated-measures ANOVA revealed a main effect for AM rate [*F*(3,27) = 37.7, *p* = 0.0001], while the *post hoc* analysis showed that the AM threshold for 300 Hz was significantly higher than those for 4 Hz (*p* = 0.0002), 40 Hz (*p* = 0.0002), and 100 Hz (*p* = 0.0002). No significant difference in AM threshold was found between 4, 40, and 100 Hz (*p* > 0.05).

### Cortical Potentials

#### AM Change: CI vs. NH

**FIGURE 2:**
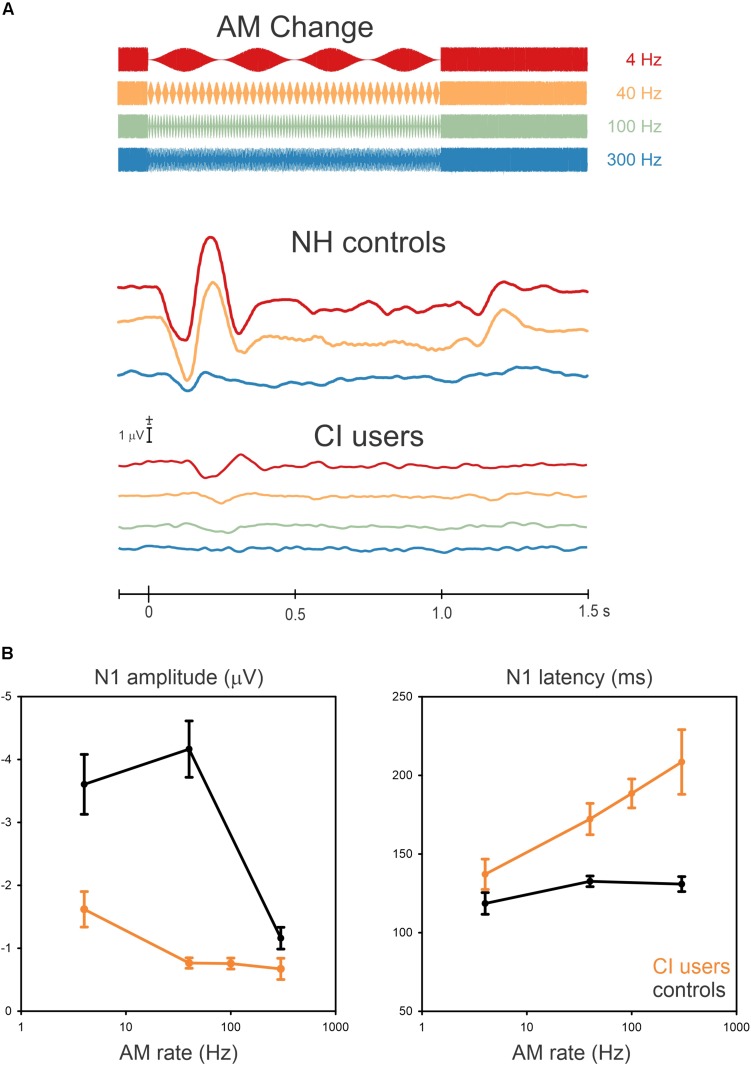
Grand mean waveforms to the AM change stimulus **(A)** and mean N1 amplitudes and latencies **(B)** are shown for NH controls and CI users. **(A)** shows responses recorded at frontal-central electrodes to the 4 (red), 40 (yellow), 100 (Green, only for CI users), and 300 Hz (blue). **(B)** shows the mean averaged N1 amplitude and latency as a function of AM rate across 10, NH and 10 CI subjects. Error bars: standard error of the mean. Overall, AM amplitudes in CI users are smaller and delayed compared to NH for all AM rates.

Grand mean data are shown in Figure 2A illustrating the cortical potentials at FC electrodes for the AM changes at 4, 40, 100, and 300 Hz with a schematic of the stimulus overlaid. The N1 responses to AM change were robust in some cases, although not all CI participants had a measurable response. The N1 responses from CI users for AM changes at the four frequencies were as follows: all of them at 4 Hz, nine at 40 Hz, eight at 100 Hz, and five at 300 Hz. The N1 responses occurred close to 150 ms after the AM change but its peak latency was prolonged with an increase in AM rate. The NH data (redrawn from Han and Dimitrijevic, 2015) shows an “off” response to the change (i.e., 100% AM change back to 0% AM) change at about 1.2 s. This was not observed in the CI data.

In general, N1 responses in the CI group decreased in amplitude and their latency was increased compared to the NH group (Figure 2B shows the N1 amplitudes and latencies as a function of AM rate for the NH and CI groups). In NH listeners, the N1 amplitude was the greatest at 40 Hz whereas the amplitudes decreased from 4 Hz to 40 Hz for CI users. In addition, the N1 latencies in the CI users were modulated as a function of AM rate, while no latency differences revealed for NH listeners.

Repeated-measures ANOVA was used to examine the effect of AM rate (4, 40, and 300 Hz) and group (CI vs. NH) for N1 amplitude and latency. For N1 amplitude, there was a significant main effect for AM rate [*F*(2,36) = 46.4; *p* < 0.0001] as well as group [*F*(1,18) = 42.5; *p* < 0.0001]. Meanwhile, the *post hoc* analysis showed that for the CI group, the N1 amplitude at 4 Hz was significantly larger than at 40 Hz (*p* = 0.007), 100 Hz (*p* = 0.007), and 300 Hz (*p* = 0.0003). Regarding the group effect, the *post hoc* testing revealed that the N1 amplitudes in the NH group were larger than the CI group (*p* = 0.0002), and for N1 latency, a significant effect of AM rate [*F*(2,36) = 23.4; *p* < 0.0001] was found such that the N1 latencies increased as the AM rate increased. The *post hoc* analysis also revealed that the N1 latency at 4 Hz was shorter than at 100 Hz (*p* = 0.001) and 300 Hz (*p* = 0.0001), while the N1 latency at 40 Hz was shorter than at 300 Hz (*p* = 0.003). No significant differences were found between 4 and 40 Hz, 40 and 100 Hz, and 100 and 300 Hz (*p* > 0.05). A significant group effect was also found for N1 latency [*F*(2,36) = 31.3; *p* < 0.0001], with the analysis showing that the N1 latencies for the CI group were delayed compared to the NH group (*p* = 0.0002).

#### AM Change: Good vs. Poor CI Performers

**FIGURE 3:**
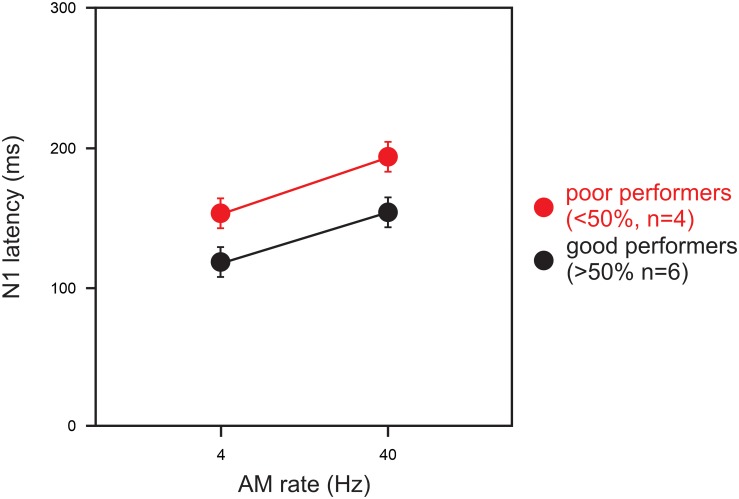
A comparison of N1 latencies between good and poor CI performers. Good performers (*n* = 6) had composite speech perception scores above 50% and poor users (*n* = 4) had scores below 50%. Note the N1 latency in poor CI performers were delayed than good CI performers for 4 and 40 Hz AM. Note that the N1 latency for 100 and 300 Hz AM were not shown since not all subject had responses for the AM rates. Errors bars: standard error of the mean.

Statistical analysis for a comparison between the good and poor CI groups was conducted for the 4 and 40 Hz AM rates only because the N1 responses at 100 and 300 Hz were not measurable in the majority of the CI subjects. For the N1 latency at 40 Hz, a significant group difference was observed such that the latencies for the good CI group were shorter than those for the poor CI group (*U* = 2.00; *p* = 0.04). Figure 3 shows the latencies for the good (*n* = 6) and poor (*n* = 4) CI performers for AM at 4 and 40 Hz. No other differences between the good and poor CI groups were found (*p* > 0.05).

### N1-Behavior Relationship

**FIGURE 4:**
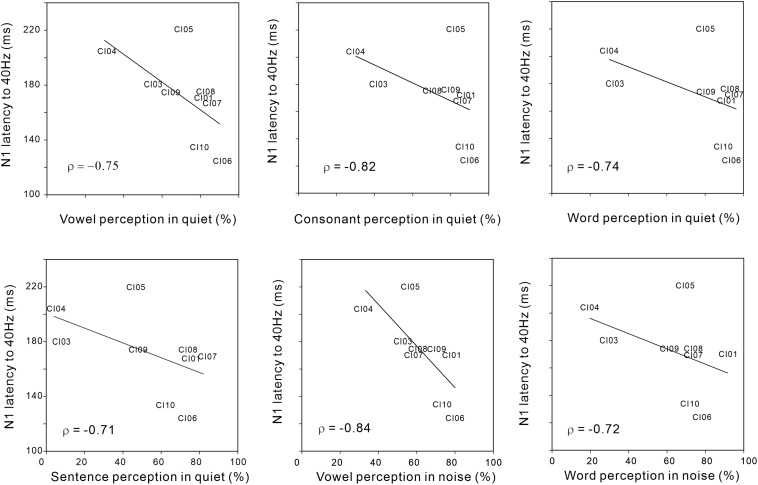
Significant Spearman correlations between N1 latency to 40 Hz AM and various speech perception measures in CI users. Note that the N1 latency to 40 Hz AM decreased as speech perception performances were better.

Figure 4 shows significant negative Spearman correlations between N1 latency for the 40 Hz AM rate and various speech perception measures including vowel (r = −0.75; *p* < 0.05), consonant (r = −0.82; *p* < 0.05), word (r = −0.74; *p* < 0.05), and sentence (r = −0.71; *p* < 0.05) perception in quiet conditions, as well as vowel (r = −0.84; *p* < 0.05) and word (r = −0.72; *p* < 0.05) perception in noise. The results indicate that shorter N1 latencies for AM at 40 Hz were associated with higher speech perception in the CI users. No significant relationships were observed for the N1 responses at different AM rates and behavioral thresholds in AM change detection (although 40 Hz AM detection threshold versus 40 Hz N1 amplitude approached significance (r = 0.59; *p* = 0.09).

Correlation analysis was also performed between N1 amplitudes/latencies and demographic variables such as subject age and duration of deafness, no significant relationships were observed.

## Discussion

The present study examined the N1 ACC-to-AM change in CI users and revealed four findings. First, although the overall N1 amplitudes were smaller for the CI group, the N1 responses to AM change were robust for low AM frequencies but less so for high ones; this pattern of N1 activity is similar to the psychoacoustic TMTFs in that the AM thresholds are low at slow AM rates and high at fast AM rates. The N1 TMTF pattern in the NH group resembled a low-pass filter shape whereas for CI users this shape was not observed. Second, N1 latency increased with an increase in AM rate. Third, for the AM rates at 4 and 40 Hz, the N1 latencies were longer for the poor CI performers compared to the good performers. Finally, there was significant correlation between the N1 latency for the AM rate at 40 Hz and speech perception.

### AM Change as a Paradigm to Assess Cortical Temporal Processing in CI Users

**FIGURE 5:**
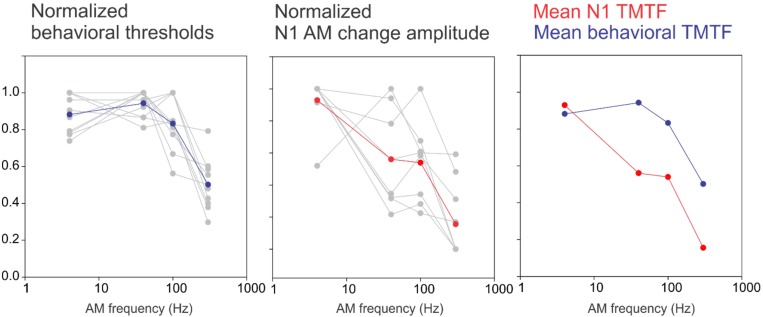
A comparison between an N1-based TMTF and a behavioral-based TMTF in CI users. For the behavioral normalization, the smallest AM detection threshold (across the four AM rates) for each subject was used as a “reference” and all other AM depth thresholds were calculated as a ratio difference from the reference. Individual normalized behavioral AM detection thresholds are shown in gray while the mean across subjects is shown in blue. A similar process was performed for N1 amplitude except that the maximum amplitude was used a reference and all other responses (at the other AM rates) were normalized as a proportion difference from the max. The middle plot shows single subjects (gray) and mean across subjects (red). The right plot compares the mean behavioral and N1 TMTFs. Note the N1 TMTF pattern in the NH resembled a low-pass filter shape whereas in CI users, the sensitivity decreased with increasing AM rate.

Previously, we developed a novel paradigm to quantify how the central auditory system encodes the detection of AM (Han and Dimitrijevic, 2015). The selected AM rates were based on timescales relevant for speech: syllables occur at slow rates near 4 Hz, formant transitions at 40–100 Hz, and fine structure near 300 Hz (Rosen, 1992). The TMTF quantifies temporal processing by measuring the ability to detect small temporal modulations in a sound as a function of AM rate. In CI users, a larger decay of the AM rate in behavioral AM thresholds has been previously observed compared to the NH control (Cazals et al., 1994; Won et al., 2011). For a direct comparison, we normalized the N1 and behavioral TMTFs in CI users using a similar approach to our previous report (Han and Dimitrijevic, 2015) and plotted the results in Figure 5. The CI behavioral TMTF resembles a low-pass filter shape similar to our previous NH data (Figure 10; Han and Dimitrijevic, 2015). However, in contrast to our previous findings in NH, the CI N1 did not have low-pass filter shape rather it continued to decrease in amplitude with increasing AM rate. The reasons for this discrepancy between the behavioral and N1 TMTF are not clear. One possibility is that they are measured differently. Behavioral TMTFs quantify the minimum AM depth needed for detection of modulation whereas the N1 response we recorded was a suprathreshold, 100% AM depth stimulus. Perhaps using AM depths closer to behavioral threshold may reveal N1 TMTF functions resembling those of behavioral TMTFs. The driving factor for the N1 TMTF low-pass filter shape in NH is that the response to 40 Hz is large and similar in magnitude to the 4 Hz response. In CI users, the 40 Hz AM change response was smaller than the 4 Hz response thus yielding a linear function. This pattern is in contrast to electrically evoked ASSRs (EASSRs) in CI users where 40 Hz responses are larger than 4 Hz (Luke et al., 2015) and represents a temporal processing difference between ASSRs and cortical N1s. The 40 Hz N1 change response, nonetheless, by itself indexes temporal sensitivity and is related to speech perception outcomes. Another potential source of the discrepancy between the shapes of the TMTF is individual variability of 40 Hz N1 response. Inspection of the normalized N1 TMTF (Figure 5) suggests that 3 CI users had a low pass filter function shape while the others had decreasing functions. However, this does not relate to individual performance (i.e., two of the three low pass filter functions came from poor performers), nor does this explain why all of the behavioral TMTFs are low-pass filter shaped. Another possibility for the behavioral-N1 TMTF discrepancy is subject state sensitivity. The behavioral TMTF requires focused attention to the stimulus whereas the N1 TMTF was recorded in a passive listening paradigm. The N1 response is known to increase with attention (Hillyard et al., 1973; Picton and Hillyard, 1974) and different N1 TMTF profiles are likely to occur with attention in CI users. This interpretation would suggest that effects of attention are differentially modulated in NH versus CI users which in itself deserves further attention.

As a subtype of temporal processing, temporal resolution includes various auditory tasks such as temporal order judgment (Tallal, 1980), gap detection (Fitzgibbons and Wightman, 2005), detection of AM (Viemeister, 1979). It is well-known that the information extracted from the temporal envelope (a slow-rate temporal component among the temporal features) is necessary for speech understanding (Rosen, 1992; Drullman et al., 1994). The temporal envelope is even more important for CI users because the CI cannot extract adequate spectral information due to a limited number of frequency channels (Shannon et al., 1995; Fu, 2002), whereas low frequency temporal information is relatively well delivered through the CI. Since behavioral studies have shown that the ability to detect temporal variations has a strong correlation with speech perception (Won et al., 2011; De Ruiter et al., 2015), there has been an effort to measure how the brain processes temporal variations using auditory-evoked responses such as the ASSR and the mismatch negativity response. Using EASSRs to AM pulse trains of 4 and 40 Hz, Luke et al. (2015) found that the EASSR amplitudes at 40 Hz were related to the AM detection thresholds in five CI users and suggested the clinical significance of EASSR as an objective measure of site-specific temporal sensitivity for CIs. Very recently, Gransier et al. (2019) found that 40-Hz EASSR variability across CI electrodes was highly correlated to speech perception in CI users. In addition, Waechter et al. (2018) found that the morphology-weighted mismatch waveform evoked by a stimulus with 8-Hz modulation is positively correlated with the AM detection threshold. Their results also suggest that cortical responses strongly follow a low-rate AM. These neurophysiological results indicate that speech perception by CI users is largely dependent on temporal information and that the auditory-evoked responses elicited by AM reflect the neuronal modulation for temporal acoustic variations. In contrast to the ASSRs, in this study, we chose to study brain responses underlying detection of AM using the N1 AM-change response.

We found that the N1 responses of the CI users decreased in amplitude as the AM rate increased to a greater degree than occurred in the NH control. In addition, the N1 latency in the CI users was almost linearly modulated as a function of AM rate, a phenomenon that was not observed in the NH group. The effect of temporal variation on N1 responses has been assessed in previous studies using various temporal features, including voice onset time (Roman et al., 2004; Dimitrijevic et al., 2013; Han et al., 2016), musical/pitch matching (Timm et al., 2012; Tan, 2017), and the temporal gap (He et al., 2018). The common finding of these studies was that the N1 response was delayed according to the delay in the onset of a sound (e.g., a long duration of voice onset time). For example, using different musical onset durations, Timm et al. (2012) found that N1 latency was longer when the onset time of a musical tone was shorter; the authors suggest that N1 latency is more sensitive to temporal change than to N1 amplitude. A recent study (Han and Dimitrijevic, 2017) examined cortical responses to varied voice onset time during passive listening also showed the linear modulation of N1 latency as a function of voice onset time. Interestingly, the more linear and consistent the N1 change with increases in voice onset time, the greater the speech perception score. This suggests that in CI users, greater sensitivity to acoustic temporal fluctuation was associated with better the speech perception outcome.

In the current study, the N1 amplitudes of the NH group were larger than those of the CI group, regardless of the AM rate. Smaller and delayed peaks are distinct characteristics of cortical responses in CI users (Beynon et al., 2005; Sandmann et al., 2009), and a decreased N1 amplitude is related to the reduced neuronal population recruited to process sounds synchronously or to how the timing and frequencies are coded at the cortex (Guiraud et al., 2007; Tremblay and Ross, 2007). However, a weak response is not always the case for CI users. Previous studies on CI use have suggested that the magnitude of cortical responses is closely related to CI speech outcomes: good CI performers revealed greater cortical responses while poor CI users attained smaller or absent peaks (Groenen et al., 1996; Kelly et al., 2005). Similarly, significant N1 latency differences between good and poor CI performers were revealed in the present study. Brain plasticity associated with hearing loss has been suggested to underlie the cortical activation pattern with hearing loss and/or with CI use (Pantev et al., 2006; Stropahl et al., 2017). However, the degree of brain plasticity can be different among CI users depending on demographic factors and environmental influences, including rehabilitation.

Although we hypothesized that the N1 TMTF would resemble the behavioral TMTF in CI users, this does not appear to be the case. The N1 response decreased with increasing AM rate suggesting neural encoding progressively decreases with faster temporal modulations. More research on the reasons for the discrepancy between behavioral and neural TMTF is warranted. This could include using AM depths closer to behavioral threshold or attentive listening paradigms.

### Cortical Responses to AM Change and Behavioral Performance in CI Users

We found that N1 latency for AM at 40 Hz was increased in the poor performing CI users compared to the good performing ones and was correlated with various speech perception measures in the CI users. Previously, it has been shown that the N1 response to simple onset sounds such as a tone burst or click is poorly related to speech perception in CI users (Firszt et al., 2002; Kelly et al., 2005). One possible explanation for this is that the N1 response is related to the detection of sound rather than its discrimination. Because speech understanding needs both detection and discrimination of sounds, many studies have focused on the cortical measures for discrimination, including mismatch negativity, P300, and ACC. Among these, ACC is evoked by changes in various stimuli such as speech (Tremblay et al., 2003; Dimitrijevic et al., 2011; Small and Werker, 2012), tone (Dimitrijevic et al., 2008, 2009), and noise (Martin et al., 1999; Han and Dimitrijevic, 2015). The ACC can be modulated as a function of frequency change and is related to the behavioral threshold for frequency discrimination (Dimitrijevic et al., 2008). In CI users, the ACC can be elicited by speech (Friesen and Tremblay, 2006; Han et al., 2016), an intensity change in the CI electrodes (Kim et al., 2009), as well as a frequency change in magnetoencephalography (Pantev et al., 2006). Moreover, the cortical responses have been successfully applied to evaluate the optimization of CI fitting in single-sided deafness (Távora-Vieira et al., 2018). These results indicate that the ACC can be reliably recorded in CI users and that the magnitude of cortical response increases with an improvement in behavioral performance. In our study, we applied the AM change paradigm to evoke the N1 ACC and attempted to correlate it with behavioral measures. The results are not surprising given that AM detection thresholds have previously shown strong correlations with various speech measures such as vowel and consonant perception (Cazals et al., 1994; Fu, 2002), phoneme perception (Xu and Zheng, 2007), and word perception (Won et al., 2011). Thus, the ACC in response to AM change can effectively reflect how the central auditory system encodes a change in AM rate, which is critical for speech understanding. This is supported by the notion that poor time-locking to the detection of the temporal envelope could be related to poor discrimination of temporal variation (Joris et al., 2004). Surprisingly, in contrast to N1 responses to frequency change (Dimitrijevic et al., 2008), no significant relationships were observed between AM behavioral thresholds and N1 latency or amplitude. Further work on AM-change-related N1/ACC responses using varying degrees of AM depth may reveal stronger relationships with behavior compared to the 100% AM depth used in the current study.

### Clinical Applications

In the present study, we showed that AM change stimuli can elicit robust cortical ACC responses (4 and 40 Hz) in CI users and the N1 latency to 40 Hz is related to speech perception measures. A larger sample of CI users is needed to determine if these findings generalize to more diverse CI populations. Interestingly only the 40 Hz N1 response showed a significant relationship with behavior while the other rates did not, even though the 4 Hz N1 response was robust. Given that behavioral TMTFs relate well to speech perception understanding in CI users, further research N1 TMTFs is warranted.

## Data Availability Statement

The datasets generated for this study are available on request to the corresponding author.

## Ethics Statement

The studies involving human participants were reviewed and approved by the Cincinnati Children’s Hospital Medical Center. The patients/participants provided their written informed consent to participate in this study.

## Author Contributions

AD: experimental design, analysis, and manuscript preparation. J-HH: analysis, experiment execution, and manuscript preparation.

## Conflict of Interest

The authors declare that the research was conducted in the absence of any commercial or financial relationships that could be construed as a potential conflict of interest.

## Footnotes

1 http://www.tigerspeech.com/
